# Be quiet and you'll keep young: does mTOR underlie p53 action in protecting against senescence by favoring quiescence?

**DOI:** 10.18632/aging.100257

**Published:** 2011-01-12

**Authors:** Vjekoslav Dulic

**Affiliations:** Institut de Génétique Moléculaire de Montpellier UMR 5535 CNRS-Université Montpellier 1 et 2, 34293 Montpellier, Cedex 5, France

Cancer is a multi-step process that involves abrogation of several barriers to uncontrolled proliferation [1]. These barriers include checkpoints that, by activating inhibitory pathways, block cell division either reversibly (quiescence) or irreversibly (senescence, apoptosis). Typically, confluence and the absence of mitogens or nutrients induce quiescence, while cell ageing, inappropriate signaling or irreparable DNA damage lead to senescence, which is characterized by a large and flat cell morphology and expression of specific biomarkers. Cell cycle arrest requires inhibition of cyclin-dependent kinases (CDK), that drive division by both activating diverse regulators involved in replication and mitosis and by inactivating pRb pocket protein family members. While pocket proteins are essential for both quiescence and senescence [2], p53, another tumor suppressor, has been thought to play a key role in senescence, mainly by inducing the CDK inhibitor p21^Waf1,Cip1,Sdi1^ (p21), which permanently blocks cell cycle progression [3]. On the other hand, although clearly involved [4], the role of p53 in quiescence is probably not essential, as this cell cycle arrest is mostly mediated by p27^Kip1^ (p27), a CDK inhibitor whose levels are not controlled by p53 [5]. Consistent with this, both serum deprivation and confluence could efficiently induce quiescence in human fibroblasts expressing the HPV16-E6 viral oncogene, which degrades p53 [6].

The straightforward role p53 in senescence has recently been challenged by Blagosklonny and colleagues, who propose that p53 is primarily a suppressor of the senescent phenotype rather than its “inducer” [7]. In agreement with growing evidence that p53 is involved in cell metabolism [4], they suggest that its another function would be to inhibit mTOR-dependent cell growth in size, thus inducing quiescence by precluding the onset of senescence. Consequently, senescence would occur in situations when the conditions for quiescence are not met and when p53 fails to suppress the mTOR pathway. This exciting but rather heretic hypothesis was based on initial observations showing that senescent phenotype requires cell growth [8,9]and that, unlike ectopic p21 expression or DNA damage by doxorubicin, p53 induction caused quiescence instead of senescence in some cells [10]. By ingeniously using a cell line in which p21 is expressed from an inducible promoter and Nutlin-3A, an Mdm2 inhibitor and potent p53 stabilizer, they showed that over-expression or stabilization of p53, prevented p21-induced senescence and instead caused quiescence. Moreover, p53 induction could “convert” p21- or oxidative stress-induced senescence into quiescence. The principal target of p53 in this case is the mTOR pathway, which plays a central role in cell size growth, as its inhibition both by nutlin 3A and rapamycin (a classical mTOR inhibitor) favours quiescence over senescence [7,11-13].

These results led to a testable hypothesis predicting that quiescence (i.e., inactive mTOR) would also prevent senescence caused by genotoxic agents. Indeed, Leontieva and Blagosklonny now show [14]that after serum-deprived or rapamycin-treated normal fibroblasts or epithelial cells have been exposed to etoposide, its removal, concomitant with serum addition, enables proliferation, indicating that quiescence compromised the onset of the senescence program. In contrast, in the continuous presence of etoposide, serum addition induced senescence, presumably by activating mTOR. Importantly, the authors showed that neither serum deprivation nor rapamycin prevented p21 induction, while the presence of DNA damage was documented by the comet assay. Lastly, they showed that, serum-deprivation prevented Nutlin 3A-induced a large, flat senescent morphology in some cancer cell lines.

Taken at face value, these results are intriguing and support the initial hypothesis. However, many questions remained unanswered. In particular, why were checkpoints not activated once cells entered the cell cycle upon serum addition (one imagines that the DNA damage caused by etoposide had not been repaired)? A possible explanation would be that truly quiescent cells were not (strongly) damaged while those that were not entirely arrested were. It is also not clear how the cells got rid of high p21 levels as they resumed cycling. Another question concerns the role of p27 in p53-induced reversible arrest. This is a highly relevant point, as mTOR inhibition blocks Akt/GSK-3-mediated p27 phosphorylation and cytoplasmic localization and leads to nuclear accumulation of p27 [15]. p27-dependent CDK inactivation together with cyclin D1 down-regulation could drive reversible cell cycle arrest together with p21 induction. Re-activation of mTOR (by serum addition) would revert this process enabling cell cycle entry, probably by degrading p27 (and p21).

The most important contribution of this and previous work is that p53 can no longer be regarded as a *bona fide* “senescence inducer” and that its role is mainly to block cell division (by stimulating p21), thus permitting the onset of senescence program under appropriate conditions [7]. In agreement with this, we showed earlier that, while in ageing human fibroblasts HPV16-E6-dependent p53 degradation failed to block DNA replication and formation of SAHFs (senescence-associated heterochromatin foci,[16]), low p53 levels did not prevent cell growth and led to a flat cell phenotype, which is also observed in senescing p21 KO MEFs [6]. Thus, senescence morphology could be uncoupled from the cell cycle arrest, which fits with the model proposed by Blagosklonny and his co-workers.
